# Impact of Maternal Parity and Direct-Fed Microbial Supplementation on Reproductive Performance, Digestibility, and Milk Quality from Early Gestation to Lactation in Sows

**DOI:** 10.3390/ani15091191

**Published:** 2025-04-22

**Authors:** Panumas Kongpanna, John A. Doerr, Uttra Jamikorn, Dachrit Nilubol

**Affiliations:** 1 Department of Animal Husbandry, Faculty of Veterinary Science, Chulalongkorn University, Bangkok 10330, Thailand; panumaskongpanna@gmail.com; 2 Agrarian Solutions, 585 Shawnee St., Nappanee, IN 46550, USA; jadoerr2@gmail.com; 3 Swine Viral Evolution and Vaccine Development Research Unit, Department of Veterinary Microbiology, Faculty of Veterinary Science, Chulalongkorn University, Bangkok 10330, Thailand

**Keywords:** digestibility, direct-fed microbials, colostrum, maternal parity, multiparous sow, reproductive performance

## Abstract

The experiment was conducted to determine the interaction effects of parity and DFM supplementation from early gestation (G 21) to lactation (L 21) on reproductive performance, apparent total tract digestibility (ATTD), colostrum IgA and IgG, and mature milk composition. Three hundred pregnant sows were blocked by parity (2, 3, 4, 5, and 6–9) and randomly assigned to two experimental diets in a randomized complete block design, with a control (CON, *n* = 150) group and direct-fed microbial (DFM, n = 150) group. The DFM contained 5 × 10^7^ cfu/g of *Bacillus subtilis* and 2 × 10^6^ cfu/g of *Lactobacillus* spp. Reproductive traits recorded included total born (TB), born alive (BA), litter weight (LW), piglets born dead (PBD) weaning weight (WW), number of weaning pig (NWP), and PWM. Two separate 14 d ATTD trials were conducted on G86 to G100 and L7 to L21. Colostrum samples were collected at 0, 3, 6, 12, and 24 h post-partum and mature milks were collected at L7 and L14 for Ig and composition analysis, respectively. Total Ig concentrations were measured by an ELISA. The interaction between diet and parity was found on LW, colostrum IgG, milk lactose, and protein (*p <* 0.05). Regardless of parity, sows fed DFM had greater reproductive performance with higher BA, LS, LW, and lower in PWM (*p <* 0.05). DFM also improved the ATTD of organic matter (OM), crude protein (CP), and ether extracts (EE) (*p <* 0.05) at G100 and gross energy (GE), CP, and EE (*p <* 0.05) at L21. Entire IgG and 3 h post-partum IgA in colostrum were higher in DFM than in the CON diet (*p <* 0.05). Parity effects were seen on NWP, LW, CP, and EE, colostrum Ig at 12 and 24 post-partum, milk protein, and lactose at L7 (*p <* 0.05). Moreover, the parity showed linear effect on TB, BA, LW, litter size (LS), WW, total PWM, the ATTD of OM and EE, colostrum IgG at 12 h and IgA at 12 and 24 h post-partum, milk fat at L7 and L21, and milk lactose at L14 (*p <* 0.05). Stepwise prediction for average colostrum IgG (mg/mL) by using nutrient digestibility = −112.97 + 0.706GE(%) + 0.518CP(%) + 0.267EE(%) (*n* = 267, R^2^ = 0.38, RSD = 6.7, *p <* 0.001). In summary, supplementing dietary DFM during early gestation through weaning had positive effects on production, the lifetime of sows, and better nutrient utilization, resulting in better milk quality and better piglet growth.

## Simple Summary

Dietary supplementation with a multi-species direct-fed microbial (DFM) (*Bacillus subtilis* and cell wall-deficient (L-form) *Lactobacillus* spp.) for multiparous sows at 21 days of gestation (G 21) to lactation (L 21) enhanced reproduction, farrowing, and litter performance and nutrient digestion. Better litter size, weight at weaning, pre-weaning mortality (PWM), colostrum immunoglobulin G (IgG), milk protein, and lactose were linearly improved, while the parity affected pigs born from DFM-fed sows with extended longevity. There was a synergistic interaction in the fifth parity of DFM sows which had the highest litter weight (LW) at weaning. Moreover, contrast results may help forecast future productivity.

## 1. Introduction

Demand for quality pork is high, making successful swine production important not only in Thailand but throughout the world. We recently reported on a very positive impact of a specialized direct-fed microbial (DFM) on various production parameters in weanling pigs [1]. While the growth of piglets is important, supplying those piglets for growth demands similar attention for sow operations. The gestation and lactation periods of sows are the critical stages in reproduction characterized by numerous changes in housing, feeding, management, and metabolism. They directly influence the lifetime productivity, longevity, and prolificacy of the sow. Intuitively, providing the appropriate nutrition throughout these stages enhances reproduction, farrowing, and litter performance.

Limiting feed intake is applied to the late gestation period to prevent gaining too much weight with resultant dystocia and poor lactation intake, subsequent reproductive failure, and insufficient milk production, especially in colostrum quality [2,3]. Colostrum quality is affected by a number of variation factors including parity, gestation length, litter weight at farrowing, and the time of first suckling of piglets [4,5,6]. Positive correlations have been identified between colostrum intake and piglet growth. Thus, optimal colostrum immunoglobulin (Ig), energy, and protein from the sows are necessary for piglet survival. Previous studies have reported that the colostrum Ig content has been influenced by parity, dropping in third-parity sows and rising in fourth- to ninth-parity sows [7]. However, as the parity number increases, so does reproductive performance, with maximized production occurring generally between the second and fifth parities and then declining thereafter [8]. Sows may be culled prior to their third or fourth parity, which is before they have achieved their most productive performance and replacement costs are covered (typically between the fourth and seventh parity) [9].

To overcome these problems, researchers have renewed interest in probiotics to limit the outbreak of drug-resistant microorganisms and for potential nutrient conservation [10]. Cell wall-deficient (L-form) *Lactobacillus* spp. have been reported for their potential probiotic activity [11]. *Bacillus subtilis* is a commonly used probiotic that can be used as an additive in animal feed. Its use alone can enhance piglet birth weight, milk production, and feed intake, with positive consequences of their nutrients for the maintenance and recovery of BW loss during lactation [12,13,14]. It tends to improve lactation intake and litter size [15]. While Bacillus is beneficial in lactating sows, multi-species DFMs can offer more. Multi-species DFMs have been shown to increase reproductive performance, WW, PWM, colostrum Ig, and milk composition [16,17,18]. In conclusion, their mode of action in the pig’s GIT includes the following key mechanisms: (1) gut microbiota modulation; (2) antimicrobial compound production; (3) extra-cellular enzyme secretion; and (4) stimulation of immunoglobulin synthesis [17]. Nevertheless, research with DFMs on reproductive performance is variable. Causes of variation include DFM strains, sow age and growth performance, ingredient composition, and inclusion rate. DFMs are often used in late gestation (G84) through lactation (L21), giving contradictory results on the effects in colostrum Ig [17,19].

However, our own experiences with DFMs led to the hypothesis that sows fed a DFM containing viable spores of *Bacillus subtilis* and cell wall-deficient (L-form) *Lactobacillus* spp. will improve reproductive performance, increase nutrient availability, and enhance maternal immunity. Therefore, this study was conducted to investigate the interaction of parity and DFM supplementation to multiparous sows from early gestation (G21) through lactation (L21) on reproductive performance, apparent total tract digestibility (ATTD), and the quantity and quality of colostrum and mature milk.

## 2. Materials and Methods

### 2.1. Direct-Fed Microbial Preparation

The multi-species DFM used in this study was a mixture of spray-dried spores of *Bacillus subtilis* and *Lactobacillus* spp. (Agrarian Solutions, Nappanee, IN, USA). This DFM contains at least 5 × 10^7^ cfu/g of *Bacillus subtilis* and 2 × 10^6^ cfu/g of *Lactobacillus* spp. The DFM rate of supplementation was 1 kg/ton fresh weight. DFM enumeration was confirmed by analyzing the feed samples.

### 2.2. Farm Management

This trial was conducted from gestation day 21 (G21) after pregnancy confirmation to lactation day 21 (L21) at a commercial pig farm in the province of Rachaburi, Thailand, with a capacity of 4500 sows. It had its own feed mill, as well as a breeding–gestation and farrowing house with partially slatted concrete floor. Gestating sows were housed in free access stalls (FASs) measuring 2.2 × 0.65 × 1.25 m (L × W × H), where each sow could enter an open stall, which would lock behind her to allow for undisturbed feeding. The stall could not be opened by other sows, and the sow could leave the stall at will. Farrowing sows were also housed in FASs measuring 2.5 × 4.0 × 1.25 m (L × W × H), equipped with guard rails, creep areas, and feed and water troughs. This FAS system allowed both gestating and lactating sows to exercise and perform natural behaviors.

This study was carried out on five consecutive sow groups from selected multiparous sows. The five batches of sows had 12.06 ± 0.73 born alive, 10.78 ± 1.15 as the number of weaned pigs, and 10.11% ± 4.48% with pre-weaning mortality during the lactation period. After artificial insemination with Duroc boar’s semen and sows were pregnant, the gestation diet (2855 kcal ME/kg or 2139 kcal NE/kg) was prepared depending on the stages of gestation. At G21, sows were fed 2.3 to 2.8 kg/sow/day; G35 to G84 were at 2.8 to 3.3 kg/sow/day and G85 to G107 at 3.3 ± 0.5 kg/sow/day. A week before farrowing, the sows were moved to the farrowing house. The feed amount was adjusted according to the body condition score to avoid an overweight condition.

At farrowing, the lactation diet (17.78% crude protein and 3064 kcal ME/kg or 2293 kcal NE/kg) begun. At the farrowing date (L1), sows were fed 0.5 kg/sow/day and progressively increased to L5 of lactation, with ad libitum access to feed from L6 until weaning. Cross-fostering was allowed only after the 24 h weighing and only within an experimental diet and parity order. Litters were standardized to 12 piglets at day 3 ± 2. After being weaned, sows were returned to the breeding–gestation house on L21. The piglets were weaned at approximately 21 days of life.

Tail docking and teeth clipping were performed once on L1 for each litter. Thereafter, daily ear tag tracking was conducted to monitor piglet numbers in each litter, ensuring accurate records of any pig movements due to cross-fostering, mortality, or removal for health reasons. Piglets received iron injections at three days of age. Commercial creep feed, presented as a 4 mm diameter short-cut pellet (3400 kcal ME/kg or 2650 kcal NE/kg, 22.21% CP) comprising corn, SBM, fish meal, whey powder, plasma protein powder, and high-amylose starch, was offered to the piglets at day 7 after birth (L7). Every morning, a creep feed was given, and feeders were checked at least twice daily and three times when providing the creep feed ad libitum. The intake of the creep feed was not recorded. Piglets had no access to sow feed before weaning.

### 2.3. Animal and Experimental Design

This observational study was conducted with 300 pregnant sows (Landrace × Large White) at G21 distributed in groups of five according to the number of parities (2nd, 3rd, 4th, 5th, and 6th to 9th; n = 30; mean parity = 4.3). Sows were allocated into two dietary treatments, one with a basal diet (CON diet, *n* = 150) and the other with basal diets supplemented with 1 kg of DFM/ton feed (DFM diet; *n* = 150).

### 2.4. Feed Sampling and Chemical Analysis

Diets were sampled prior to the start and end of the trial and submitted for proximately analysis by Animal Nutrition Laboratory (Department of Animal Husbandry, Faculty of Veterinary Science, Chulalongkorn University, Thailand) according to the method described in the AOAC [20] and Van Soest et al.’s study [21] for determinations of dry matter (DM), organic matter (OM), crude protein (CP), ether extract (EE), crude fiber (CF), starch, calcium and phosphorus, and neutral detergent fiber (NDF) and acid detergent fiber (ADF).

Feed samples were dried to a constant weight at 60 °C using a forced-air oven (UF110, MEMMERT, GmbH, Schwabach, Germany). Dried samples were milled to pass a 0.5 mm screen before analysis, and all analyses were carried out in triplicate (Ultra Centrifugal Mill ZM 200, Retsch, GmbH, Schwabach, Germany). The test ingredients were analyzed for gross energy (GE) using bomb calorimetry (C 6000; IKA-Werke GmbH & Co., Staufen, Germany). Dry matter (DM, procedure 930.15) in the test samples were analyzed by using a forced-air oven (UF110, MEMMERT, GmbH). Ash (procedure 942.05) was determined after ignition of a known weight of diets and feces in a furnace (Ney Vulcan 3-1750 Box Furnace, CNSI, Los Angeles, CA, USA) at 500 °C. Nitrogen was determined using the Kjeldahl method with a Kjeltec (Kjeltec TM 2200, Foss Tecator, Hillerød, Denmark). These samples were analyzed for CP content (nitrogen × 6.25; procedure 988.05).

Ether extract (EE; procedure 920.39) was analyzed using an accelerated solvent extraction system (SoxtecTM 8000, FOSS, Hillerød, Denmark). Samples were then loaded onto the extraction system and extracted under elevated temperature (120 °C) and pressure using petroleum ether as the extraction solvent.

Crude fiber (CF; prodecure 962.09), neutral detergent fiber (NDF), and acid detergent fiber (ADF) were analyzed as described by Van Soest et al. [21] using a raw fiber extractor (FIWE6, VELP Scientifica Inc., Usmate Velate, Italy).

Starch was analyzed by using 0.1 mL of α-amylase and 0.1 mL of amyloglucosidase (Megazyme International Ireland Ltd., Wicklow, Ireland) according to AOAC-991.43 and AACC-33-07.01 methods [20,22].

Calcium in the diet samples was analyzed using an atomic absorption spectrometer (Z-2000, Hitachi, Tokyo, Japan). Total P concentration was determined using a UV–visible spectrophotometer (U-1000, Hitachi, Tokyo, Japan).

The formulation and nutrient specifications of the gestation and lactation sow diet (mash form) are shown in Table 1. The basal diet was formulated to meet or exceed National Research Council [23] requirements of nutrient standards for gestation and lactation in sows. All other nutrients met or exceeded the NRC recommendations during the whole gestation and lactation period.

### 2.5. Field-Recorded Variables

The following reproductive data were recorded: farrowing rate, gestation and lactation length, wean to first service interval (WSI), total born (TB), born alive (BA), stillborn piglets, mummified fetuses, litter weight (LW), individual piglet body weight, and the number of weaned piglets. The litter weight and pre-weaning mortality (PWM) rate were also recorded weekly. From birth to weaning, the survival rate and the weaning weight of piglets of each group were recorded and analyzed. Body condition score was evaluated using a scale ranging from 1 (emaciated) to 5 (grossly obese), combining visual appraisal and palpation at specific body points.

### 2.6. Digestibility Trial

To perform apparent total tract digestibility (ATTD), chromic oxide (Cr_2_O_3_) was added as a marker to an experimental diet at 0.3% feed as fed. The gestation and lactation digestibility trial was conducted on G86 to G100 and L7 to L21, respectively. Both trials were investigated for 2 weeks following an adaptation period, and collection was carried out on the first and second weeks, respectively. Fecal samples were collected from the floor twice daily, pooled within sows and the collection period, and stored at −20 °C until analysis.

Experimental diet and feces samples were freeze-dried (beta 1-8 LSCbasic freeze dryer, MARTIN CHRIST, GmbH, Schwabach, Germany) and finely ground to pass through a sieve of 1 mm mesh size prior to analyses. Diet and fecal samples were analyzed in duplicate for nutrient chemical composition according to the AOAC [20] and Van Soest et al. [21], as mentioned above. The intra-assay coefficient of variability (%CV) threshold of less than 3% was used for GE, DM, OM, CP, and EE, and it was less than 5% for NDF and ADF.

An atomic absorption spectrophotometer, AAS, (Perkin Elmer 3110) (Perkin Elmer, Waltham, MA, USA) was used to determine Cr_2_O_3_ with a Perkin Elmer lamp (Perkin Elmer, MA, USA) for Cr (part #303-6021 Serial H235571). The standard for AAS from J.T. Baker (Phillipsburg, NJ, USA) of 1000 µg/mL CAS 6449-04 was used for chromium.

The ATTD was calculated by the indicator method using Cr contents in the diet and fecal samples. The formula to calculate ATTD is

ATTD (%) = (1 − (Cr_diet_/Cr_digesta_) × (Nutr_digesta_/Nutr_diet_)) × 100 (1)

where Cr_diet_ is the Cr concentration in the diet, Cr_digesta_ is the Cr concentration in the feces, Nutr_digesta_ is the nutrient content in the feces, and Nutr_diet_ is the nutrient content in the diet [24].

### 2.7. Colostrum and Mature Milk

Before milking, all teats were cleaned with ethanol wipes and sterile gloves were worn. Colostrum (3 mL) was collected within the day after first piglet parturition (L1) without the use of oxytocin to represent 0 h and then at 3, 6, 12, and 24 h post-partum. Mature milk (5 mL) was collected at L7 and L14. All samples were placed in conical tubes and subdivided and stored at −80 °C until further analysis.

Milk yield was determined at L1, L7, and L14 using the weigh–suckle–weigh method [25] by removing the piglets from the sow and weighing each piglet individually using a precise scale. Record these initial weights (P1). Return the piglets to the sow for nursing. Allow them to nurse for a predetermined time, typically about 24 h. Remove the piglets from the sow and weigh them again (P2). Milk yield can now be calculated as

Milk yield (kg/day) = litter weight after suckling (P2) − litter weight before suckling (P1)

Colostrum and milk samples were analyzed for chemical composition (protein, fat, lactose, total solids, and somatic cell count (SCC)) after adding 0.1 mL of azidiol as a preservative (containing 12 mg sodium azide and 0.5 mg chloramphenicol per 100 mL of milk) using the Milko-Scan FT 6000 (Foss Electric, Hillerød, Denmark).

Colostrum samples were stored at −80 °C for analyzed immunoglobulin G (IgG, MBS777719) and A (IgA, MBS1600873) concentration by using a commercial ELISA kit (Pig Ig ELISA KIT, Mybiosource, San Diego, CA, USA). The CV was 3.04 and 3.22 for colostrum IgG and IgA, respectively. The detection range was 7.81–500 and 15.6–1000 ng/mL for IgA and IgG assays, respectively. The ELISA was read using a microplate reader (AccuReader; Model M965/M965+, Metertech, Nangang, Taipei, Taiwan, ROC).

### 2.8. Statistical Analysis

Statistical analyses were performed as a randomized complete block design (RCBD) with 2 treatments, using the parity as the block and sows as the experimental unit. The categorical variable was the parity with the following distribution: (parity 2, *n* = 30; parity 3, *n* = 30; parity 4, *n* = 30; and parities 6 to 9, *n* = 30). Data were analyzed using a *t*-test to compare differences between CON and DFM. An advanced mixed model using the GLM procedure was used for an analysis of variance (ANOVA) for RCBD. The significance of differences was calculated using Duncan’s multiple range test (SAS Institute Inc., Cary, NC, USA).

Parity effects were partitioned into linear and quadratic components using orthogonal polynomial contrasts. Non-normal distributions such mortality data were analyzed using the GLIMMIX procedure of SAS software (Version 9.4) for generalized linear mixed models. The normality of data was tested by graphic (P-P, Q-Q plots) and numerical (Shapiro–Wilk test) methods. A Duncan multiple range significance test was then carried out when *p* was <0.05. Those included in the models are reported in the following model:

*Yij* = μ + α*i* + β*j* + αβ*ij* + ε*ijl* (2)

where *Yij* is the parameter for the observations.

μ is the mean of the observations.

α*i* is the effect of the experimental diets (CON and DFM).

β*j* is the effect of parity (2nd, 3rd, 4th, 5th, and 6th to 9th).

αβ*ij* is the interaction effect between the experimental diets and the parity number.

ε*ijl* is the random error.

For the repeated measurements in the above models (digestibility trial, litter performance, colostrum Ig, and milk composition at different time points), sows were added into the model as a random effect to account for repeated measurements within sows by using the variance component as the covariate. Thus, the final model was

Y*ijk* = μ + α*i* + β*j* + αβ*ij* + δ*k* + ε*ijkl* (3)

δ*k* is the effect of the individual sows (sow_1_, sow_2_, sow_3_, ……, sow_29_, sow_30_).

Stepwise regression analyses were developed using PROC REG (SAS Institute Inc., Cary, NC, USA), and predictor variables were assessed with a backward stepwise procedure in which the *p <* 0.10 variables were added into the model.

All numerical data are presented as means ± standard deviation (SD).

## 3. Results

### 3.1. Reproductive and Farrowing Performance

The effects of parity and DFM supplementation and their interaction on reproductive and farrowing performance are presented in Table 2.

The reproductive performance parameters were not significantly influenced by DFM supplementation (*p >* 0.05) in terms of the farrowing rate, lactation length, total born (TB), and percentage of mummified fetuses. A significant decrease in gestation length (115.1 vs. 115.5 d, *p <* 0.035), weaning to first serving interval (WSI) (7.14 vs. 7.94 d, *p <* 0.012), piglets born dead (PBD) (3.97 vs. 7.14%, *p <* 0.042), and stillborn (1.54 vs. 3.69%, *p <* 0.027) and an increase in born alive (born alive) (12.52 vs. 11.60 n, *p <* 0.010), litter weight (16.48 vs. 19.43 kg, *p <* 0.001), and birth weight (1.45 vs. 1.57 kg, *p <* 0.044) were observed in the DFM group compared to the CON.

**Figure 1:**
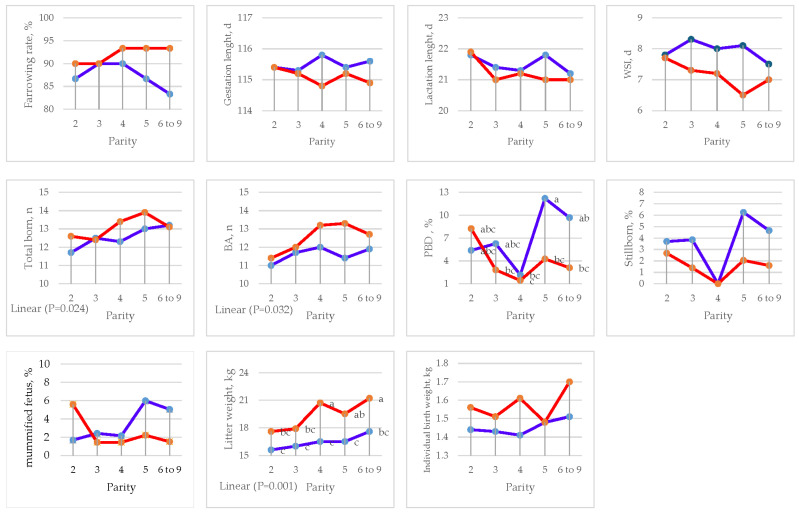
Effect of sow parity on farrowing performance in multiparous sows fed CON and DFM diets (blue line = CON diet; red line = DFM diet). ^a,b,c^ Within a line, least squares means without a letter in common are significantly different (*p <* 0.05). WSI = wean to first service interval; BA = born alive; PBD = piglets born dead.

Parity had an effect on PBD (*p <* 0.009) and litter weight (*p <* 0.003) with linear contrast (*p <* 0.001). Moreover, linearity was found in TB (*p <* 0.024) and BA (*p <* 0.032) (Figure 1 and Table 3).

No interaction between treatment and parity was observed for the reported dependent variables.

### 3.2. Litter Performance

The effects of parity and DFM supplementation and their interaction on litter performance and pre-weaning mortality (PWM) are presented in Table 4.

The litter performance parameters were not significantly influenced by DFM supplementation (*p >* 0.05) in PWM at L14 and L21. A significant increase in litter weight (*p <* 0.01) at L7, L14, and L21, the number of piglets at L7, L14, and L1, body weight at L7, L14, and L21, and PWM at L7 was observed in the DFM group compared to the CON.

**Figure 2:**
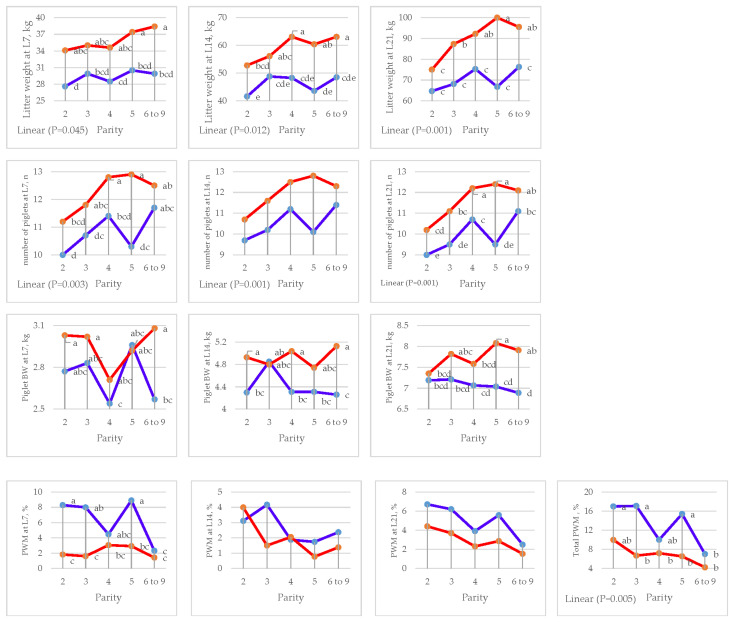
Effect of sow parity on litter performance and pre-weaning mortality (PWM) in multiparous sows fed CON and DFM diets (blue line = CON diet; red line = DFM diet). ^a,b,c,d,e^ Within a line, least squares means without a letter in common are significantly different (*p <* 0.05). PWM = pre-weaning mortality.

Parity had an effect on litter weight at L7 (*p =* 0.046), L14 (*p =* 0.029), and L21 (*p =* 0.001) with linear contrast, the number of piglets at L7 (*p =* 0.012), L14 (*p =* 0.001), and L21 (*p =* 0.001) with linear contrast, and total PWM (*p =* 0.032) with linear contrast (Figure 2 and Table 5).

There was an interaction (*p =* 0.046) between treatment and parity for litter weight at L21 in the fifth parity of sows fed DFM, which had the highest litter weight, while sows fed the DFM diet with third to ninth parity had a significantly higher litter size (*p <* 0.001) when compared with sows fed the CON diet. Likewise, a trend (*p =* 0.089) was found for the number of piglets at L21.

### 3.3. Nutrient Digestibility

The effects of parity and DFM supplementation and their interaction on the ATTD of gestation and lactation results are reported into Table 6.

The ATTD parameters were not significantly influenced by DFM supplementation (*p >* 0.05) in GED, DMD, NDFD, and ADFD in the gestation period and GED, DMD, OMD, NDFD, and ADFD in the lactation period. A significant increase in the ATTD of OMD (80.37 vs. 78.45%, *p =* 0.005), CPD (73.07 vs. 70.10%, *p =* 0.001), and EED (77.30 vs. 71.27%, *p =* 0.001) in the gestation period was observed in the DFM group compared to the CON. Similarly, there was a significant increase in the ATTD of CPD (76.17 vs. 73.05%, *p =* 0.001) and EED (79.31 vs. 66.86%, *p =* 0.001) in the lactation period.

**Figure 3:**
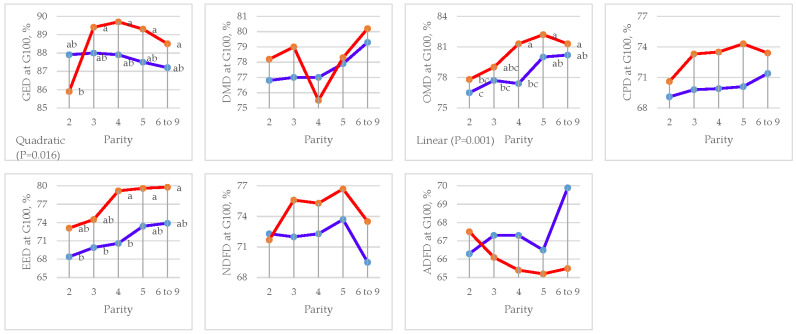
Effect of sow parity on apparent total tract digestibility (ATTD) in gestation period in multiparous sows fed CON and DFM diets (blue line = CON diet; red line = DFM diet). ^a,b,c^ Within a line, least squares means without a letter in common are significantly different (*p <* 0.05). GE = gross energy; DM = dry matter; OM = organic matter; CP = crude protein; EE = ether extract; NDF = neutral detergent fiber; ADF = acid detergent fiber.

**Figure 4:**
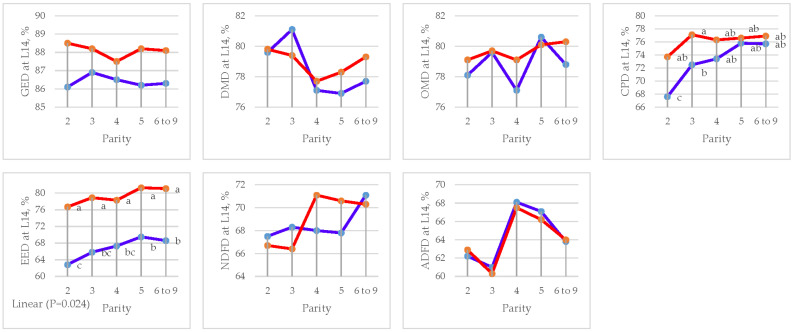
Effect of sow parity on apparent total tract digestibility (ATTD) in lactation period in multiparous sows fed CON and DFM diets (blue line = CON diet; red line = DFM diet). ^a,b,c^ Within a line, least squares means without a letter in common are significantly different (*p <* 0.05). GE = gross energy; DM = dry matter; OM = organic matter; CP = crude protein; EE = ether extract; NDF = neutral detergent fiber; ADF = acid detergent fiber.

Parity had an effect on OMD (*p =* 0.002) with linear contrast and EED (*p =* 0.024) in the gestation period and CPD (*p =* 0.001) and EED (*p =* 0.010) with linear contrast in the lactation period (Figure 3 and Table 7). Moreover, quadratic contrast (*p =* 0.016) was found in GED in the gestation period (Figure 4 and Table 8).

No interaction between treatment and parity was observed for the reported dependent variables. A trend (*p =* 0.087) was found for GED at the gestation period.

### 3.4. Colostrum Immunoglobulin

The effects of parity and DFM supplementation and their interaction on colostrum IgG and IgA are presented in Table 9.

IgA was not significantly influenced by DFM supplementation (*p >* 0.05) in colostrum IgA at 0, 6, 12, and 24 h post-partum. IgG, however, was significantly increased (*p =* 0.001) at 0, 3, 6, 12, and 24 h (103 vs. 86, 97 vs. 83, 71 vs. 63, 32 vs. 24, and 32 vs. 24 mg/mL, respectively). Concentrations of colostrum IgA were greater (*p =* 0.005) in sows fed the DFM diet than the CON at 3 h post-partum (19 vs. 15 mg/mL)

**Figure 5:**
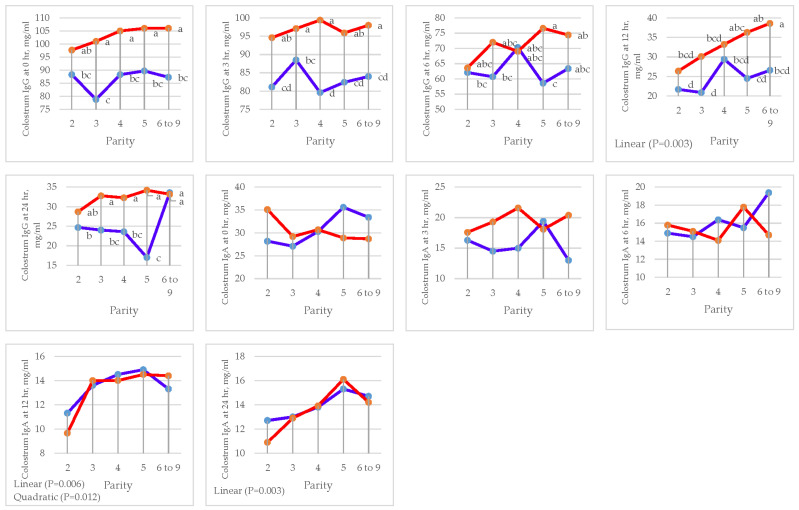
Effect of sow parity on colostrum immunoglobulin (Ig) G and A in multiparous sows fed CON and DFM diets (blue line = CON diet; red line = DFM diet). ^a,b,c,d^ Within a line, least squares means without a letter in common are significantly different (*p <* 0.05).

Parity had an effect on colostrum IgG at 12 h (*p =* 0.045) by linear contrast. There were also parity effects on colostrum IgA at 12 h (*p =* 0.011) by linear and quadratic contrast and at 24 h (*p =* 0.030) by linear contrast (Figure 5 and Table 10).

There was an interaction (*p =* 0.013) between treatment and parity in sows fed the CON diet, with fifth parity having the lowest colostrum IgG concentration at 24 h post-partum, while sows fed the DFM diet with the third to ninth parity had a significantly higher colostrum IgG concentration (*p <* 0.001). However, no interaction between treatment and parity was observed for colostrum IgA concentration.

### 3.5. Milk Yield and Composition

The effects of parity and DFM supplementation on milk yield and composition are presented in Table 11 for lactation days 1, 7, and 14, respectively.

A significant increase in milk fat (5.42 vs. 5.06%, *p =* 0.02), protein (10.82 vs. 9.84%, *p =* 0.002), SNF (18.21 vs. 17.18%, *p =* 0.008), and total solids (23.63 vs. 22.25%, *p =* 0.001) at L1 was observed in the DFM group compared to the CON. At L7, a significant increase in milk protein (4.56 vs. 4.08%, *p =* 0.015), SNF (11.31 vs. 10.15%, *p =* 0.001), and total solids (16.33 vs. 14.95%, *p =* 0.001) was observed in the DFM group compared to the CON. Similarly, at L14, a significant increase in milk yield (10.83 vs. 10.17 kg, *p =* 0.012) and fat (4.14 vs. 3.75, *p =* 0.001) and a reduction in SCC (4182 vs. 4810 SCC/mL, *p =* 0.02) were observed in the DFM group compared to the CON.

**Figure 6:**
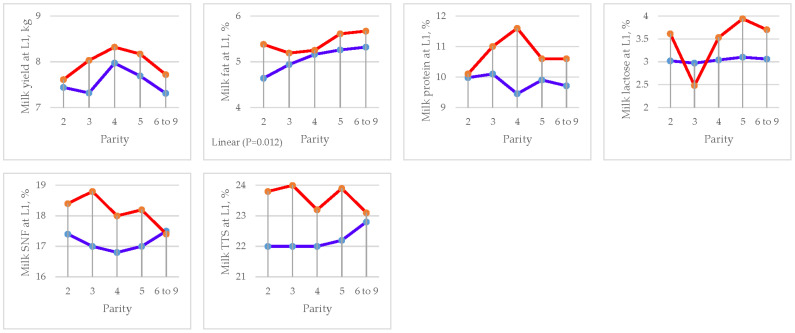
Effect of sow parity on milk yield and composition at L1 in multiparous sows fed CON and DFM diets (blue line = CON diet; red line = DFM diet).

**Figure 7:**
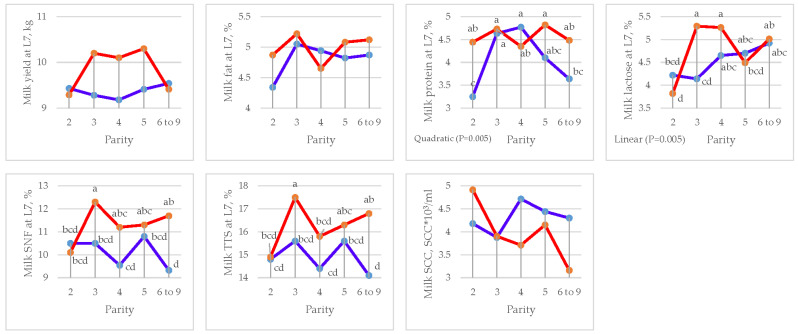
Effect of sow parity on milk yield and composition at L7 in multiparous sows fed CON and DFM diets (blue line = CON diet; red line = DFM diet). ^a,b,c,d^ Within a line, least squares means without a letter in common are significantly different (*p <* 0.05).

**Figure 8:**
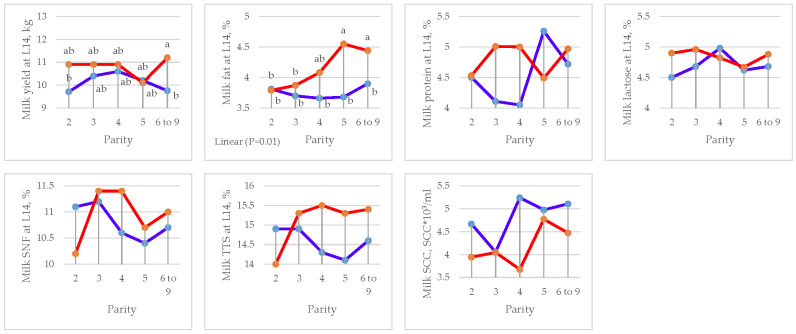
Effect of sow parity on milk yield and composition at L14 in multiparous sows fed CON and DFM diets (blue line = CON diet; red line = DFM diet). ^a,b^ Within a line, least squares means without a letter in common are significantly different (*p <* 0.05).

Parity affected milk protein at L7 (*p =* 0.041) by quadratic contrast and lactose at L7 (*p =* 0.003) by linear contrast. Linear effects were found in milk fat composition (L1) (*p =* 0.012), lactose (L7) (*p =* 0.006), and fat (L14) (*p =* 0.011). Effects by quadratic contrast were found in milk protein (L7) (*p =* 0.005) and total solids (L7) (*p =* 0.019) (Figure 6, Figure 7 and Figure 8 and Table 12, Table 13 and Table 14).

There was an interaction (*p =* 0.013) between treatment and parity for milk lactose at L7 (*p =* 0.026) and milk protein at L14 (*p =* 0.038).

### 3.6. Using Stepwise Regression to Estimate Colostrum IgG Concentration

Predictor variables used in the analysis were the nutrient digestibility of gestation sows. The results of the predictions of colostrum IgG concentration (mg/mL) based on gestation nutrient digestibility profiles are presented in Table 15.

### 3.7. Multi-Regression Models to Predict Colostrum IgG Concentration in Post-Partum Period

The prediction of colostrum IgG concentration from an equation was the average IgG (mg/mL) = −112.97 + 0.706GE(%) + 0.518CP(%) + 0.267EE(%), which had an R^2^ of 0.38, RSD = 6.7 (*p <* 0.001) (Table 16).

## 4. Discussion

### 4.1. Sow’s Nutritional Status

Our study shows that dietary DFMs increased energy utilization during lactation. This may indicate an elevation of energy retention by the sows. Energy is a key component in sows’ nutritional status. A negative correlation between gestational energy intake and lactation feed intake has been reported [26], and while high energy may increase the ovulation rate, it may also lower the survival of embryos [27]. In this study, DFMs shortened weaning to first service interval (7.94 ± 1.38 vs. 7.14 ± 1.70 days), and 3064 kcal/kg ME proved to be an appropriate energy level to avoid the culling of sows, since low gestational energy can negatively affect milk production [28].

In addition, balance between energy and protein is important. Protein that is too high during gestation can reduce feed efficiency. It has been shown that raising CP from 13% to 21% causes an increase in farrowing time (8 h vs. 4.5 h) and metabolic costs to synthesize urea from excess protein [29]. Conversely, Mahan and Grifo [30] reported that decreasing the crude protein level of the lactation diet from 16% or 18% to 12% or 14% resulted in reduced feed consumption and consequently reduced weight gain in the sows and lower litter weights. Lactating sows require more energy reserves and nutrients to sustain body tissues and support milk production. Our study showed that dietary supplementation with DFMs significantly increased energy utilization during the lactation period, suggesting that the energy retention of sows was elevated and that a moderate CP level for gestation (14.54%) and lactation (17.78%) had a positive effect on sows’ performance during the farrowing and lactating periods.

### 4.2. Direct-Fed Microbial Effect

The scientific literature offers little information on the efficacy in DFM-supplemented sows for a long term under commercial husbandry conditions. The present data show that such supplementation in multiparous sows improved reproductive, farrowing, and litter performance by increasing energy and nutrient digestibility, as well as improving colostrum immunoglobulin and milk composition.

The voluntary feed intake of sows in the early lactation period is associated with a commercial goal to achieve milk production in order to support large litters with a minimal utilization of sow body reserves. Although lactating sows’ intakes were not measured in this study, an increase in litter weight, piglets’ body weight, and a reduction in the pre-weaning mortality rate appear related to improved colostrum Ig and milk composition concentrations.

Sows that lose excessive body condition scores (BCSs) during lactation experience significant reproductive challenges, as evidenced by multiple studies; they have a longer weaning to first serving interval (WSI), indicated by a non-reproductive day extension [31]. Providing DFMs to sows led to significant reductions in WSI. A shorter WSI, i.e., decreased duration of estrus and the interval from onset of estrus to ovulation, may improve performance. Sows mated within 5 days will show better fertility and prolificacy rates than those mated in 6–10 days [32].

Total born piglets were not affected by DFM supplementation, probably resulting from genetics, age at conception, nutrition, lactation length, parity distribution, boar contribution, management, etc. [33]. In the current study, five standardized groups of multiparous sows had 12.06 ± 0.73 of BA, 10.78 ± 1.15 of NWP, and 10.11% ± 4.48% of PWM during the lactation period. DFM sows had significantly higher (10 to 20%) BA, LW, and birth weight and fewer stillborn piglets than the CON.

These results differ from other studies, where the DFM containing 1.0 × 10^8^ CFU/kg *B. subtilis* PB6 and fed from G90 until weaning (L21) yielded lower birth weights than the controls [14,34]. However, we note that the initiation of DFM was 90 days into gestation, while this study began supplementation at day 21 of gestation. G90 DFM studies (fetal growth) fail to find performance effects of DFMs because farrowing and litter effects are determined at G60 (placental growth) [35].

In commercial swine herds in Thailand, the percentage of stillborn varies from 5.3 to 8.7% between herds of large white × landrace [33,36,37]. This study demonstrated that sows fed a DFM diet had only 1.54% of stillborn during the farrowing period. It has been proven that older sows are more probable to having a high stillbirth rate and extended farrowing, potentially as a result of weak uterine contractions [33]. During parturition, many sows experience anorexia and can refuse eating for the next 48 h. Large litter size is also a factor that affects this value. Overall, energy deficiency will contribute to prolonging farrowing duration, stillborn piglets, the irregular uniformity BW of piglets, and mortality. Maternal DFM supplementation can boost energy retention by being more efficient in digestion and preventing energy deficiency during parturition [38]. Similarly, this suggests that providing timely parturition assistance can reduce the number of stillborn piglets by half.

The results of digestibility in this study indicated that the DFM can significantly improve the ATTD of GE, OM, CP, and EE. A better gestational nutrient digestibility of DM, CP, and EE improves reproductive performance as placental and fetal growth improve [39]. As nutrient digestion during gestation improves, so does placenta and fetal growth. Better nutrient digestion during lactation improves milk quality and induces better growth and weaning weights, as shown in this study.

The present study shows that the supplementation of DFM during pregnancy and lactation improved colostrum IgG and increased the percentage of milk fat, protein, TS, SNF, and SCC that all influence the regulation of the immune response and immune complex mammary cell function. The results of this study were similar to those by Ayala et al. [13], who reported that the inclusion of *B. subtilis* in lactating sows affected milk production and the concentration of IgG. Jang et al. [40] showed that the supplementation of DFM stimulates colostrum IgG synthesis, which was strongly correlated with the presence of immunoglobulin in the serum of piglets. Over 50% of the cells in sow colostrum and milk are phagocytes, which promote healthy intestinal mucosa and prevent infections in the early post-partum period [41]. Babicz et al. [42] reported that sows with lower SCC values had higher milk yields and litters with a higher BW and daily weight gain. The presence of leukocytes (white blood cells) and epithelial cells in milk is reflected in SCC. Elevated SCC levels frequently represent an immunological reaction to infections, including mastitis, which may damage the mammary gland [41].

### 4.3. Parity Effect

Sow parity can affect reproductive performance throughout productive life. Third to fifth parity sows gave the highest litter birth weight and weaning weight and the lowest PBD and PWM, IgG, milk protein, and lactose production. The parity of sows had no effect on BA, that being associated with the number of ovulations and embryo survival [43]. This study also confirms no effect of parity on stillborn and mummified fetuses despite parity being significantly related to the risk of intrapartum stillborn [44]. The present study observed that parity was only associated with increased litter weight.

The strong negative correlation between birth weight and stillborn rate is conflicting [45]. Birth weight within-litter variation is also affected by parity; older sows had a higher risk to produce low-uniformity litters, as well as a higher proportion of low-birth-weight piglets [46]. Lightweight piglets in large litters could be related to growth delay given smaller glycogen pools which lower competition with large littermates for milk consumption [47].

Pre-weaning mortality is, on average, 11.0% in pigs [48], similar to the level of 13.35% in this study. Contrary to our results, Milligan et al. [49] reported that second- and third-parity sows experienced reduced PWM and consequently weaned more piglets per litter than younger sows, whereas we found that the fourth parity had less PMW and more piglets per litter than younger sows. Older-parity sows could have a high piglet born dead count due to many BA piglets, which indicates sows having a longer farrowing duration. Koketsu et al. [50] also suggest that a higher PWM is linked to having more piglets, having more stillborn piglets, having shorter gestation periods, and the farrowing season.

Parity affects colostrum yield [51]. However, there are no sow parity data on colostrum concentration at 24 h after parturition in the present study. Typically, older sows had greater milk yield than younger sows, which results in older sows raising heavier pigs to weaning [52]. Additionally, older sows have more and better developed mammary glands that produce more milk compared to younger sows [53].

Numerous factors affecting milk yield, regardless of parity, could be litter size, time to first suckling, and the measurement method. According to the correlations found between milk yield, the average piglet growth rate, and litter size, the number of functioning glands is the primary determinant of milk yield [54]. The time of first suckling was significantly related to colostrum yield and mammary development [55]. Milk yield can be measured as either single [25] or whole point [56].

Neonatal piglets receive maternal immunity via colostrum. Piglets from younger sows are more likely to get sick than those from older sows, because the milk from younger sows does not have as many antibodies from their colostrum [57]. In contrast, from an immunological perspective, the sow’s parity did not affect the concentration of immunoglobulins in the colostrum [54]. Although there has been very little research into the effect of parity on colostrum Ig, in our study, colostrum IgG was significantly influenced by parity at 24 h post-partum. The concentration of milk and plasma cytokines is linked to a sow’s parity. Available cytokines and colostrum in mammary glands from the sow make the colostrum better for the immune system, which has an immunomodulatory effect and is a strong driver of reproduction and their offspring growth [58,59]. Our study revealed no effect of parity on the IgA concentration, which is in agreement with Amatucci et al. [2].

There is an average of 3.1–4.6 parity-based removal rate for sows in commercial herds in the United States, with udder issues, low productivity, and old age being the most common reasons for removal in older-parity sows [9]. In Thailand, culling is performed in seventh-parity sows, which was derived from reproductive disorders (20–30%), old age (15–20%), low productivity (10–15%), and lameness (5–10%).

### 4.4. Interaction Effect Between Maternal Parity and DFM

Analyses indicated an interaction between DFM and maternal parity that contributed to the litter weight (LW) at L21, colostrum IgG at 24 h post-partum, milk protein, and lactose. The synergistic interaction affected the fifth-parity DFM sows, which had the highest LW at weaning compared with the average of the other parities. In addition, this interaction affected the fourth-parity CON sows, which had the highest milk protein at L14 compared with the average of the other parities. This result indicated that fifth-parity sows had ability to integrate and cooperate with dietary DFMs improving energy retention during the lactation period, as well as quality milk production.

Antagonistic interaction affected the fifth-parity CON sows, which had the lowest colostrum IgG content compared with the average of the other parities due to age-related physiological and immunological changes [2,4]. There were similar effects on the second-parity DFM sows, which had the lowest milk lactose content.

Sows in the fourth and sixth to ninth parity were positively influenced by the inclusion of DFMs, resulting in higher litter weight at weaning. This result indicated that colostrum IgG and milk protein and lactose in the older sows positively influences litter weight [56].

Another potential explanation for the increased litter weaning weight of older sows is that the birth weight is higher, resulting in stronger piglets better able to reach and stimulate the udder to produce more milk [60]. Similarly, heavier piglets also consume more milk and provide better udder stimulation [61].

### 4.5. Stepwise Regression to Estimate Colostrum IgG Concentration

Stepwise regression indicated that the nutrient digestibility of GE, CP, and EE affected colostrum IgG concentration. This suggests that the supplementation of DFM during the early gestation period increased nutrient digestibility, which improved the colostrum IgG concentration and, potentially, increased the survival of the piglets. Energy, protein, and fat produced substrates for colostrogenesis. The colostrum proteins not only supply amino acids to the neonate but also bioactive factors, enzymes, and immunoglobulin [62].

Stepwise regression can verify the adequacy of colostrum for piglets to lower the risk of PWM throughout their lives. It is a practical way to predict colostrum IgG at the herd level. However, the Brix refractometer is sensitive enough for use at the farm level as well [61]. Regardless, nutrient digestion can be predicted at G90, providing information in sufficient time before farrowing to develop strategies to improve colostrum consumption. This would seem to be a more efficient management approach than measuring colostrum IgG concentration post-parturition, which is rarely performed due to the difficulty in measuring the amount of colostrum produced.

### 4.6. Orthogonal Contrasts

Linear trends in our study included TB, BA, and LW. Better values of these metrics increased linearly as the parity increased. Similar results occurred in the number of weaned piglets (n), OM and EE digestibility (%), IgG and IgA concentrations (mg/mL) at 12 and 24 h post-partum, and fat and lactose content in milk (%). The total PWM (%) trend was negatively linear, suggesting that older sows provided maternal privilege and quality milk for their piglets better than younger sows. Older sows were associated with higher BW piglets, suggesting a greater capacity to digest and utilize nutrients as well as synthesize immunoglobulin and produce colostrum [2,4,58]. Due to their older age and previous maternal experience, multiparous sows are more physiologically, behaviorally, and immunologically mature than younger sows, although their udder morphology and structure are less desirable in terms of accessibility for the piglets [63].

Quadratic patterns represent the most accurate description of commercial practice of parity distribution. Based on this study, GE digestibility, IgA, and milk protein decreased quadratically as the parity increased. It is a widely accepted theory that when a sow reaches maturity, a downward quadratic trend will be seen, which may also reflect the sow’s age and biological response to colostrum IgA and protein synthesis. Lower colostrum IgA at the end of farrowing increases neonatal piglet diarrhea and risk of death before weaning while affording them a slight weight gain [64]. In addition, milk protein provides essential and nonessential amino acids for muscle development in suckling piglets as well. Collectively, the regulation of these traits could be considered as a risk factor of being culled, providing researchers an opportunity for identifying and evaluating factors related to sow culling.

In the present study, a lower piglet body weight (pBW) at L7 was produced at the fourth and sixth parities in sows fed the CON diet; in contrast, sows receiving DFMs had a better recovery of their body weight. A possible explanation for these quadratic trends is that older sows may have a carry-over effect on reproductive performance and maternal manner, but they also may have inadequate energy during the transition period, especially if feed-restricted to control body condition [48,58,64].

Additionally, the lower pBW at L7 but not litter weight (LW) at L7 was most likely related to the number of piglets at L7. Sows at the fourth and sixth parities always maintained over 11 piglets, while only 10 piglets were found at the fifth parity of sows fed the CON diet. Thus, a higher number of piglets had competition among littermates. In this study, a higher number of PWM showed that approximately 20% of deaths results from starvation or the crushing of weak, malnourished, and young piglets. Moreover, rearranging piglets from a teat face double costs: they expend energy in finding and obtaining a teat and they often fail to obtain milk during early nursing periods.

Finally, the correlation between pBW and milk intake suggests that older, bigger piglets can consume greater quantities of milk from the mammary glands of lactating sows. Thus, lower milk production in older sows may result from a smaller udder, consequently limiting milk intake and litter growth. Positive correlations have been identified between colostrum intake and piglet growth. Thus, greater amounts of colostrum immunoglobulin, energy, and protein provided by the sows are necessary for piglet survival. Although the multiparous sow in this study is not considered as part of the hyperprolific breeding goals yet, the results show that it is possible to breed for an increased litter size and litter weight at weaning. Both of these traits can provide economic and long-term benefits for piglet production.

The number of PSY is dependent upon the number of BA, PWM, and fertility between consecutive farrowing for the sow. Both the piglet’s birth weight and the quantity of energy stored as lipids and glycogen are impacted by the sow’s nutrition and management throughout the early stages of pregnancy. Supplementing dietary DFMs in the early stages of gestation, modifying a sow’s metabolism to allocate additional nutrients to the fetus, are strategies for enhancing piglet birth weight and energy reserves. As nutrient digestion during pregnancy improves, so does placental and fetal growth. Properly nourishing the sow during lactation is crucial for optimizing milk output and energy, which influences piglet survival to weaning and the sow’s reproductive performance post-weaning.

Consequently, this study emphasized the potential for strategic feeding to enhance the sow’s performance in subsequent lactation. A proper nutritional management of gestating sows is a key issue to maximize the number of piglet weaned/year and to optimize sow longevity.

## 5. Conclusions

In conclusion, the supplementation of multi-species DFMs exerted a beneficial effect on their farrowing performance, improved nutrient digestion and, consequently, enhanced colostrum, milk protein, and fat synthesis. Reproductive performance at the fifth parity is reduced for older parities; however, efficiency can be raised through DFM supplementation, which affects maximizing productivity and reducing unnecessary culls.

Interaction data indicated that DFM supplementation significantly improved the reproductive performance of younger sows and extended the lifetime production of older sows by producing sufficient amounts of colostrum to meet the requirements of piglet survival and the growth rate. Furthermore, we assert that this innovative study will offer a novel perspective of DFMs on sow longevity with appropriate parity management.

## Figures and Tables

**Table 1:** Ingredient composition and analyzed chemical composition of gestation and lactation diet and creep feed.

Ingredient Composition, %	Gestation	Lactation	Creep Feed
Broken rice	30.25	39.92	-
Extruded corn	-	-	25.5
Cassava powder	10.94	-	-
Whey powder	-	-	15.5
Rice bran oil	6.76	1.41	-
Plasma protein powder	-	-	10.0
Rice bran	20.42	25.14	15.0
Soybean protein concentrate	-	-	19.0
Soy bean meal 46%	13.97	13.97	-
Full fat soy bean meal	13.85	14.67	-
Lactose	-	-	5.0
Potato protein	-	-	2.5
Egg powder	-	-	2.5
Vegetable oil	-	-	5.0
L-lysine	0.15	0.23	-
DL-methionine	0.09	0.09	-
Monodicalcium phosphate P2	1.89	2.49	-
Calcium carbonate	1.09	1.49	-
Salt	0.29	0.29	-
Premix for pregnant sows ^1^	0.30	-	-
Premix for lactating sows ^2^	-	0.30	-
Premix for piglets	-	-	1.00
Chemical composition	% as fed		
DM	90.87	90.04	90.24
OM	92.47	91.16	92.42
CP	14.54	17.78	22.21
EE	8.72	7.88	0.85
CF	5.4	4.4	2.50
NDF	11.22	11.79	-
ADF	5.44	5.49	-
Starch	36.12	38.76	-
GE, Kcal/kg	3300	3550	4210
ME, Kcal/kg ^3^	2855	3064	3400
NE, Kcal/kg	2139	2293	2650
Calcium	0.169	0.167	0.71
Phosphorus	0.724	0.666	0.65

^1^ Provided the following amount of 12.5% Ca, 3.6% P, 3.2% Na, 1.2% Lys, 0.3% Met, 1% Thr, 1.6 MIU vitamin A; 0.32 MIU vitamin D; 7.0 g vitamin E; 0.33 g vitamin K3; 1.00 vitamin B2; 0.0005 vitamin B12; 6.7 g nicotinic acid; 3.0 g pantothenic acid; 0.034 g biotin; 34 g copper; 40 g ferrous sulfate; 0.25 iodine; 11 g Mn; 25 g zinc; 0.084 Se; 1 kg carrier. ^2^ Provided the following amount of 13.3% Ca, 4.5% P, 3.3% Na, 4% Lys, 1.1% Met, 1.6% Thr, 1.6 MIU vitamin A; 0.32 MIU vitamin D; 7.0 g vitamin E; 0.33 g vitamin K3; 1.00 vitamin B2; 0.0005 vitamin B12; 6.7 g nicotinic acid; 3.0 g pantothenic acid; 0.034 g biotin; 34 g copper; 40 g ferrous sulfate; 0.25 iodine; 11 g Mn; 25 g zinc; 0.084 Se; 1 kg carrier. ^3^ Metabolizable energy (ME) and net energy (NE) are formulated values (EvaPig^®^), and other nutrient levels are measured values.

**Table 2:** Interaction effect of parity and DFM supplementation on reproductive and farrowing performance.

		Treatment ^1^		Source of Variation ^2^			Contrast ^3^	
		CON	DFM	TRT	PAR	TRT × PAR	L	Q
Mated sow	n	150	150					
Farrowed sow	n	131	138					
BCS at G90		2.98 ± 0.37	2.94 ± 0.53					
BCS at L1		3.04 ± 0.12	3.13 ± 0.21					
BCS at L21		2.55 ± 0.29	2.78 ± 0.19					
Farrowing rate	%	87.33 ± 2.79	92.00 ± 1.83	0.190	0.974	0.922	1.000	0.500
Gestation length	day	115.50 ± 1.05	115.10 ± 0.76	0.036	0.987	0.384	0.707	0.849
Lactation length	day	27.10 ± 0.76	27.02 ± 0.82	0.619	0.418	0.777	0.722	0.293
Wean to first service (WSI)	day	7.94 ± 1.38	7.14 ± 1.70	0.013	0.723	0.639	0.178	0.819
Total born	n	12.54 ± 1.94	13.08 ± 1.82	0.154	0.195	0.741	0.025	0.521
Born alive	n	11.60 ± 1.83	12.52 ± 1.75	0.011	0.110	0.606	0.032	0.106
Litter weight	kg	16.48 ± 2.04	19.43 ± 3.14	0.001	0.003	0.535	0.001	0.990
Birth weight	kg/n	1.45 ± 0.28	1.57 ± 0.28	0.045	0.625	0.780	0.291	0.308
Piglets born dead	%	7.14 ± 8.72	3.97 ± 7.16	0.043	0.009	0.176	0.606	0.129
Stillborn	%	3.69 ± 6.07	1.54 ± 3.24	0.028	0.084	0.666	0.675	0.145
Mummified fetuses	%	3.46 ± 5.15	2.43 ± 5.24	0.319	0.525	0.133	0.696	0.341

^1^ CON, control diet based on soybean meal diet; DFM, control + 1.0 kg of DFM contains 5 × 10^7^ cfu/g of *Bacillus subtilis* and 2 × 10^6^ cfu/g of *Lactobacillus* spp. ^2^ Source of variation: TRT = treatment; PAR = parity effect; and TRT × PAR = interaction. ^3^ L = linear and Q = quadratic. G90 = day 90 of gestation period; L1 and L21 = day 1 and day 21 of lactation period.

**Table 3:** The regression equation of a sow’s parity order and variable on reproductive and farrowing performance.

Variable (s)	Contrast	Treatment	Equation	Square of the Correlation (R^2^)
Total born, n	Linear (*p =* 0.024)	CON	y = 0.35x + 11.49	R^2^ = 0.8676
		DFM	y = 0.25x + 12.33	R^2^ = 0.4257
Born alive, n	Linear (*p =* 0.032)	CON	y = 0.15x + 11.15	R^2^ = 0.3409
		DFM	y = 0.39x + 11.35	R^2^ = 0.5788
Litter weight, kg	Linear (*p =* 0.001)	CON	y = 0.88x + 16.74	R^2^ = 0.7426
		DFM	y = 0.45x + 15.09	R^2^ = 0.8992

**Table 4:** Interaction effect of parity and DFM supplementation on litter performance and pre-weaning mortality (PWM).

			Treatment ^1^		Source of Variation ^2^			Contrast ^3^	
			CON	DFM	TRT	PAR	TRT × PAR	L	Q
Litter weight	Day 7	kg	29.33 ± 4.75	35.94 ± 6.46	0.001	0.283	0.918	0.046	0.916
	Day 14	kg	46.21 ± 8.57	59.13 ± 10.54	0.001	0.029	0.530	0.013	0.210
	Day 21	kg	69.92 ± 10.33	90.08 ± 16.29	0.001	0.001	0.046	0.001	0.097
Number of piglets	Day 7	n	10.82 ± 1.48	12.24 ± 1.72	0.001	0.012	0.397	0.003	0.201
	Day 14	n	10.52 ± 1.43	11.98 ± 1.57	0.001	0.001	0.249	0.001	0.097
	Day 21	n	9.96 ± 1.28	11.60 ± 1.47	0.001	0.001	0.089	0.001	0.079
Piglet body weight	Day 7	kg/n	2.74 ± 0.42	2.95 ± 0.45	0.013	0.114	0.348	0.617	0.398
	Day 14	kg/n	4.41 ± 0.62	4.93 ± 0.52	0.001	0.564	0.101	0.716	0.867
	Day 21	kg/n	7.08 ± 0.65	7.75 ± 0.89	0.001	0.738	0.357	0.582	0.555
PWM ^4^	Day 7	%	6.39 ± 6.73	2.17 ± 3.54	0.001	0.148	0.229	0.164	0.327
	Day 14	%	2.65 ± 4.47	1.94 ± 3.61	0.388	0.433	0.705	0.089	0.407
	Day 21	%	4.98 ± 6.61	2.96 ± 5.18	0.098	0.358	0.990	0.065	0.963
	Total	%	13.35 ± 10.19	6.93 ± 6.57	0.001	0.032	0.472	0.005	0.784

^1^ CON, control diet based on soybean meal diet; DFM, control + 1.0 kg of DFM contains 5 × 10^7^ cfu/g of *Bacillus subtilis* and 2 × 10^6^ cfu/g of *Lactobacillus* spp. ^2^ Source of variation: TRT = treatment; PAR = parity effect; TRT × PAR = interaction. ^3^ L = linear and Q = quadratic. ^4^ PWM = pre-weaning mortality.

**Table 5:** The regression equation of sow’s parity order and variable on litter performance and pre-weaning mortality (PWM).

Variable (s)	Contrast	Treatment	Equation	Square of the Correlation (R^2^)
Litter weight at L7, kg	Linear (*p =* 0.045)	CON	y = 0.52x + 27.72	R^2^ = 0.4754
		DFM	y = 1.1x + 32.6	R^2^ = 0.8497
Litter weight at L14, kg	Linear (*p =* 0.012)	CON	y = 0.86x + 43.58	R^2^ = 0.1667
		DFM	y = 2.49x + 51.63	R^2^ = 0.7526
Litter weight at L21, kg	Linear (*p =* 0.001)	CON	y = 2.19x + 63.67	R^2^ = 0.4383
		DFM	y = 5.37x + 73.89	R^2^ = 0.7849
Number of piglets at L7, n	Linear (*p =* 0.003)	CON	y = 0.3x + 9.92	R^2^ = 0.4352
		DFM	y = 0.37x + 11.13	R^2^ = 0.6544
Number of piglets at L14, n	Linear (*p =* 0.001)	CON	y = 0.33x + 9.53	R^2^ = 0.4977
		DFM	y = 0.44x + 10.66	R^2^ = 0.6846
Number of piglets at L21, n	Linear (*p =* 0.001)	CON	y = 0.42x + 8.7	R^2^ = 0.5526
		DFM	y = 0.51x + 10.07	R^2^ = 0.7517
Total PWM, %	Linear (*p =* 0.005)	CON	y = −2.168x + 19.806	R^2^ = 0.5663
		DFM	y = −1.163x + 10.423	R^2^ = 0.8047

**Table 6:** Interaction effect of parity and DFM supplementation on apparent total tract digestibility (ATTD) in gestation and lactation periods.

			Treatment ^1^		Source of Variation ^2^			Contrast ^3^	
			CON	DFM	TRT	PAR	TRT × PAR	L	Q
Gestation	GE ^4^	%	87.77 ± 1.95	88.60 ± 2.07	0.09	0.090	0.088	0.356	0.016
	DM	%	77.65 ± 4.09	78.28 ± 3.92	0.552	0.331	0.864	0.208	0.166
	OM	%	78.45 ± 2.68	80.37 ± 3.01	0.005	0.002	0.661	0.001	0.391
	CP	%	70.10 ± 3.56	73.07 ± 2.92	0.001	0.347	0.831	0.765	0.903
	EE	%	71.27 ± 5.57	77.30 ± 5.43	0.001	0.024	0.886	0.070	0.845
	NDF	%	72.00 ± 5.48	74.62 ± 4.89	0.062	0.429	0.837	0.331	0.243
	ADF	%	67.51 ± 5.85	65.97 ± 6.15	0.352	0.967	0.873	0.452	0.300
Lactation	GE	%	86.46 ± 2.79	88.14 ± 2.22	0.019	0.992	0.972	0.792	0.948
	DM	%	78.54 ± 3.53	78.94 ± 3.55	0.667	0.223	0.803	0.121	0.317
	OM	%	78.88 ± 3.63	79.72 ± 2.55	0.320	0.462	0.888	0.348	0.964
	CP	%	73.05 ± 4.82	76.17 ± 2.48	0.001	0.001	0.246	0.894	0.813
	EE	%	66.86 ± 4.44	79.31 ± 4.17	0.001	0.010	0.925	0.024	0.779
	NDF	%	68.56 ± 4.67	69.05 ± 4.35	0.675	0.257	0.528	0.327	0.546
	ADF	%	64.47 ± 7.81	64.21 ± 6.27	0.887	0.103	0.998	0.573	0.352

^1^ CON, control diet based on soybean meal diet; DFM, control + 1.0 kg of DFM contains 5 × 10^7^ cfu/g of *Bacillus subtilis* and 2 × 10^6^ cfu/g of *Lactobacillus* spp. ^2^ Source of variation: TRT = treatment; PAR = parity effect; TRT × PAR = interaction. ^3^ L = linear and Q = quadratic. ^4^ GE = gross energy; DM = dry matter; OM = organic matter; CP = crude protein; EE = ether extract; NDF = neutral detergent fiber; ADF = acid detergent fiber.

**Table 7:** The regression equation of sow’s parity order and variable on apparent total tract digestibility (ATTD) in gestation period.

Variable (s)	Contrast	Treatment	Equation	Square of the Correlation (R^2^)
GED at G100, %	Quadratic (*p =* 0.016)	CON	y = −0.0786x^2^ + 0.2814x + 87.72	R^2^ = 0.9727
		DFM	y = −0.6643x^2^ + 4.4957x + 82.38	R^2^ = 0.9114
OMD at G100, %	Linear (*p =* 0.001)	CON	y = 0.97x + 75.45	R^2^ = 0.8638
		DFM	y = 1.02x + 77.26	R^2^ = 0.7679

**Table 8:** The regression equation of sow’s parity order and variable on apparent total tract digestibility (ATTD) in lactation period.

Variable (s)	Contrast	Treatment	Equation	Square of the Correlation (R^2^)
EED at L14, %	Linear (*p =* 0.024)	CON	y = 1.53x + 62.21	R^2^ = 0.8427
		DFM	y = 1.12x + 75.9	R^2^ = 0.8279

**Table 9:** Interaction effect of parity and DFM supplementation on colostrum immunoglobulin (Ig) G and A.

			Treatment ^1^		Source of Variation ^2^			Contrast ^3^	
			CON	DFM	TRT	PAR	TRT × PAR	L	Q
IgG	0 h	mg/mL	86.54 ± 8.21	103.47 ± 9.43	0.001	0.154	0.459	0.059	0.768
	3 h	mg/mL	83.18 ± 6.47	97.04 ± 6.58	0.001	0.405	0.360	0.654	0.645
	6 h	mg/mL	63.06 ± 11.01	71.17 ± 10.08	0.004	0.529	0.162	0.172	0.403
	12 h	mg/mL	24.67 ± 9.11	32.97 ± 7.64	0.001	0.045	0.597	0.004	0.541
	24 h	mg/mL	24.62 ± 8.45	32.29 ± 4.87	0.001	0.028	0.013	0.082	0.155
IgA	0 h	mg/mL	30.95 ± 11.05	30.55 ± 11.77	0.896	0.932	0.638	0.794	0.757
	3 h	mg/mL	15.67 ± 4.71	19.44 ± 5.29	0.005	0.792	0.185	0.772	0.374
	6 h	mg/mL	16.18 ± 3.74	15.54 ± 4.07	0.524	0.555	0.187	0.152	0.489
	12 h	mg/mL	13.56 ± 3.37	13.34 ± 3.13	0.778	0.011	0.850	0.006	0.012
	24 h	mg/mL	13.93 ± 3.12	13.64 ± 3.15	0.711	0.030	0.860	0.004	0.231

^1^ CON, control diet based on soybean meal diet; DFM, control + 1.0 kg of DFM contains 5 × 10^7^ cfu/g of Bacillus subtilis and 2 × 10^6^ cfu/g of Lactobacillus spp. ^2^ Source of variation: TRT = treatment; PAR = parity effect; TRT × PAR = interaction. ^3^ L = linear and Q = quadratic.

**Table 10:** The regression equation of sow’s parity order and variable on colostrum immunoglobulin (Ig) G and A.

Variable (s)	Contrast	Treatment	Equation	Square of the Correlation (R^2^)
Colostrum IgG at 12 h, mg/mL	Linear (*p =* 0.003)	CON	y = 1.34x + 20.6	R^2^ = 0.3653
		DFM	y = 3.06x + 23.74	R^2^ = 0.9937
Colostrum IgA at 12 h, mg/mL	Quadratic (*p =* 0.012)	CON	y = −0.5929x^2^ + 4.0871x + 7.78	R^2^ = 0.5903
	Linear (*p =* 0.006)	DFM	y = 0.996x + 10.326	R^2^ = 0.9849
Colostrum IgA at 24 h, mg/mL	Linear (*p =* 0.003)	CON	y = 0.63x + 12.01	R^2^ = 0.8167
		DFM	y = 0.98x + 10.66	R^2^ = 0.6633

**Table 11:** Interaction effect of parity and DFM supplementation on milk yield and composition.

		Treatment ^1^		Source of Variation ^2^			Contrast ^3^	
		CON	DFM	TRT	PAR	TRT × PAR	L	Q
Day 1								
Milk yield	kg	7.54 ± 0.66	7.97 ± 1.14	0.092	0.228	0.973	0.787	0.073
Fat	%	5.06 ± 0.59	5.42 ± 0.58	0.021	0.170	0.730	0.012	0.888
Protein	%	9.84 ± 1.11	10.82 ± 1.21	0.003	0.748	0.328	0.878	0.254
Lactose	%	3.04 ± 0.71	3.45 ± 1.08	0.083	0.253	0.413	0.265	0.553
Solid not fat	%	17.18 ± 1.46	18.21 ± 1.35	0.009	0.857	0.606	0.384	0.788
Total solids	%	22.25 ± 1.70	23.63 ± 1.25	0.001	0.960	0.658	0.894	0.761
Day 7								
Milk yield	kg	9.37 ± 0.84	9.89 ± 1.20	0.062	0.744	0.433	0.728	0.262
Fat	%	4.81 ± 0.58	4.99 ± 0.66	0.263	0.290	0.602	0.295	0.470
Protein	%	4.08 ± 0.91	4.56 ± 0.71	0.015	0.041	0.079	0.767	0.005
Lactose	%	4.52 ± 0.57	4.77 ± 0.88	0.134	0.003	0.026	0.006	0.093
Solid not fat	%	10.15 ± 1.41	11.34 ± 1.50	0.002	0.215	0.115	0.969	0.314
Total solids	%	14.95 ± 1.66	16.33 ± 1.52	0.001	0.067	0.293	0.645	0.217
Somatic cell count	10^3^/mL	4.30 ± 1.20	3.96 ± 1.23	0.292	0.495	0.323	0.279	0.969
Day 14								
Milk yield	kg	10.17 ± 1.07	10.83 ± 0.92	0.013	0.529	0.301	0.838	0.409
Fat	%	3.75 ± 0.43	4.15 ± 0.49	0.001	0.089	0.117	0.011	0.539
Protein	%	4.53 ± 0.78	4.80 ± 0.77	0.163	0.586	0.038	0.180	0.790
Lactose	%	4.69 ± 0.68	4.85 ± 0.62	0.377	0.905	0.877	0.965	0.677
Solid not fat	%	10.85 ± 1.58	10.99 ± 1.49	0.732	0.770	0.794	0.772	0.554
Total solids	%	14.59 ± 1.45	15.13 ± 1.55	0.180	0.835	0.445	0.642	0.671
Somatic cell count	10^3^/mL	4.81 ± 0.99	4.18 ± 1.06	0.020	0.261	0.390	0.058	0.752

^1^ CON, control diet based on soybean meal diet; DFM, control + 1.0 kg of DFM contains 5 × 10^7^ cfu/g of *Bacillus subtilis* and 2 × 10^6^ cfu/g of *Lactobacillus* spp. ^2^ Source of variation: TRT = treatment; PAR = parity effect; TRT × PAR = interaction. ^3^ L = linear and Q = quadratic.

**Table 12:** The regression equation of sow’s parity order and variable on milk yield and composition at L1.

Variable (s)	Contrast	Treatment	Equation	Square of the Correlation (R^2^)
Milk fat at L1, %	Linear (*p =* 0.012)	CON	y = 0.168x + 4.56	R^2^ = 0.9154
		DFM	y = 0.1x + 5.12	R^2^ = 0.5495

**Table 13:** The regression equation of sow’s parity order and variable on milk yield and composition at L7.

Variable (s)	Contrast	Treatment	Equation	Square of the Correlation (R^2^)
Milk protein at L7, %	Quadratic (*p =* 0.005)	CON	y = −0.3207x^2^ + 1.9493x + 1.758	R^2^ = 0.8705
		DFM	y = −0.0293x^2^ + 0.1927x + 4.308	R^2^ = 0.0923

**Table 14:** The regression equation of sow’s parity order and variable on milk yield and composition at L14.

Variable (s)	Contrast	Treatment	Equation	Square of the Correlation (R^2^)
Milk fat at L14, %	Linear (*p =* 0.01)	CON	y = 0.016x + 3.702	R^2^ = 0.0615
		DFM	y = 0.198x + 3.552	R^2^ = 0.858

**Table 15:** Stepwise regression to estimate colostrum IgG concentration (mg/mL) based on gestation nutrient digestibility profiles.

Variable ^1^	Unit	Estimate	*p*-Value	Selected Variable
Intercept	Constant	−112.97	0.014	−112.97
Gestation GE	%	0.706	0.086	0.706
Gestation DM	%	0.163	0.407	-
Gestation OM	%	0.298	0.266	-
Gestation CP	%	0.518	0.031	0.518
Gestation EE	%	0.267	0.048	0.267
Gestation NDF	%	0.208	0.137	-
Gestation ADF	%	0.058	0.638	-

N = 267. ^1^ GE = gross energy; DM = dry matter; OM = organic matter; CP = crude protein; EE = ether extract; NDF = neutral detergent fiber; ADF = acid detergent fiber.

**Table 16:** Multiple regression equation to predict colostrum IgG concentration (mg/mL) based on gestation nutrient digestibility profiles.

Dependent Variable	Unit	R-Square	*p*-Value	Equation ^1^
Average IgG	mg/mL	0.38	0.001	−112.97 + 0.706GE(%) + 0.518CP(%) + 0.267EE(%)
IgG 0 h post-partum	mg/mL			Average IgG (mg/mL) × 30.74%
IgG 3 h post-partum	mg/mL			Average IgG (mg/mL) × 29.22%
IgG 6 h post-partum	mg/mL			Average IgG (mg/mL) × 21.71%
IgG 12 h post-partum	mg/mL			Average IgG (mg/mL) × 9.21%
IgG 24 h post-partum	mg/mL			Average IgG (mg/mL) ×9.13%

^1^ GE = gross energy; CP = crude protein; EE = ether extract.

## Data Availability

The data that support the findings of this study are available upon re-quest from the corresponding author.

## Abbreviations

The following abbreviations are used in this manuscript:

ADF	Acid detergent fiber
ATTD	Apparent total tract digestibility
BA	Born alive
BW	Body weight
CON	Control group
CP	Crude protein
DFM	Direct-fed microbial
DM	Dry matter
EE	Ether extract
G	Gestation day
GE	Gross energy
IgA	Immunoglobulin A
IgG	Immunoglobulin G
L	Lactation day
LS	Litter size
LW	litter weight
LW	Litter weight
ME	Metabolic energy
NDF	Neutral detergent fiber
NE	Net energy
NWP	Number of weaning pigs
OM	Organic matter
PBD	Piglets born dead
pBW	Piglet body weight
PWM	Pre-weaning mortality
RSD	Residual standard deviation
TB	Total born
WEI	Weaning to estrous interval
WSI	Weaning to first service interval
WW	Weaning weight
